# Effect of Grain Refinement on the Comprehensive Mechanical Performance of W–Cu Composites

**DOI:** 10.3390/nano13030386

**Published:** 2023-01-18

**Authors:** Tielong Han, Chao Hou, Yaochuan Sun, Yurong Li, Xiaoyan Song

**Affiliations:** Faculty of Materials and Manufacturing, Key Laboratory of Advanced Functional Materials, Ministry of Education of China, Beijing University of Technology, Beijing 100124, China

**Keywords:** W–Cu composites, high-temperature performance, wear resistance, grain refinement

## Abstract

W–Cu composites are commonly subjected to coupled multiple fields in service, which imposes high requirements on their overall performance. In this study, the ultrafine-grained W–Cu composite was fabricated using the combination of electroless plating and spark plasma sintering. The wear resistance and high-temperature compressive properties of the ultrafine-grained W–Cu composite were investigated and compared with those of the commercial coarse-grained counterpart. Moreover, the underlying strengthening and wear mechanisms were also discussed. Here we show that the ultrafine-grained W–Cu composite exhibits superior integrated mechanical performance, making it a potential alternative to commercial W–Cu composites.

## 1. Introduction

Tungsten–copper (W–Cu) composites are typical bimetal materials composed of immiscible tungsten and copper, which inherit the advantages of both tungsten and copper, such as high strength and hardness, excellent wear and arc erosion resistance, good electrical and thermal conductivity, etc. [1,2]. It is widely used in vacuum contacts for high-voltage switches, resistance welding electrodes, and electronic packaging [3,4,5,6]. It is also a candidate material for the electromagnetic gun rails and the high-temperature heat sink material in future fusion reactors [7,8]. Therefore, W–Cu composites have attracted increasing attention in the past decades due to their excellent integrated properties and multi-functional applications.

Significantly, W–Cu composites are generally in service under multi-field coupling conditions, which imposes high requirements on their overall performance [9]. For instance, as electrical contact materials, excellent arc erosion and wear resistance, high strength in elevated temperatures, and good conductivity are needed [10]. An adjustable thermal expansion coefficient, good conductivity, and high-temperature strength are required for heat sink materials [11]. As a result, any performance degradation would affect the service reliability and durability of W–Cu composites due to the cask effect. Therefore, a systematic study of the global properties of W–Cu composites is necessary to assess their potential for applications.

It should be emphasized that W–Cu composites have to bear harsh wear processes and high-temperature loads in numerous service conditions [12,13]. Therefore, both wear resistance and high-temperature characteristic are essential indicators for high-end applications of W–Cu composites [14]. However, most studies to date have mainly focused on investigating the hardness and strength of W–Cu composites at room temperature [15,16,17,18,19,20,21]. For the enhancement of wear resistance and high-temperature strength of W–Cu composites, grain refinement and second-phase strengthening are commonly used methods [22,23]. Owing to the high thermal stability and superior high-temperature properties, WC has attracted much attention as the strengthening phase of conventional W–Cu composites [12,24,25,26]. Zhang et al. fabricated WC-reinforced W–25Cu composites using the in-situ reaction of graphite and tungsten, and they found that the high-temperature strength of W–Cu composites was significantly improved due to the formation of WC [12]. Similarly, Chen et al. also elevated the high-temperature strength of W–Cu composites with the introduction of WC [24]. In addition, it has also been reported that the wear resistance of W–Cu composites in a vacuum can be dramatically improved by adding WC [14]. Nevertheless, the addition of WC usually resulted in a non-negligible drop in electrical conductivity, which was unsatisfactory. Moreover, grain refinement is beneficial for the wear resistance and high-temperature properties of W–Cu composites. It was reported the fine-grained W–Cu composites with a grain size of ~1 μm showed higher high-temperature strength than the commercial counterparts with a grain size of ~8 μm [27]. In addition, ultrahigh high-temperature strength was achieved in nanostructured W–Cu–Cr–ZrC composites [28]. The wear resistance of nanostructured W–Cu composites was also largely improved [29]. Unfortunately, a large number of phase interfaces in nanocrystalline W–Cu also cause a decrease in electrical conductivity [29]. Therefore, achieving the combined enhancement of wear resistance and high-temperature strength with a nonsignificant decrease in conductivity remains challenging.

In our previous work [30], ultrafine-grained (UFG) W–Cu composites were prepared using electroless plating and spark plasma sintering. Well-balanced room-temperature compressive strength and electrical conductivity were achieved using the combination of grain refinement with a continuous copper network structure. However, other properties of the UFG W–Cu composite still remain to be explored. Accordingly, in this paper, the friction behavior and high-temperature compressive performance were further investigated, which were then compared with those of the commercial coarse-grained (CCG) W–Cu composite. This work sheds light on the effects of grain refinement on wear resistance and high-temperature properties as well as the underlying mechanisms, which are of great importance for the design of advanced metal matrix composites.

## 2. Experimental

### 2.1. Materials and Methods

Ultrafine tungsten powders with an average particle size of ~240 nm and a purity of 99.9% (Hawk, Beijing, China) were used as the raw material. All the other reagents used in this paper were analytically pure. In this work, the UFG W–Cu composite with a copper content of 30 wt.% was chosen as a representative due to its common use. For the fabrication of the UFG W–Cu composite, tungsten powders were firstly coated with copper using a modified electroless plating process; more details can be found in our previous work [30]. Then, the obtained copper-coated tungsten powders were reduced at 800 °C for 60 min in a H_2_ atmosphere to remove oxide. Thereafter, the composite powders were consolidated using spark plasma sintering (SPS) at 1000 °C for 5 min under a pressure of 100 MPa. The heating rate was 100 °C/min, and the specimens were cooled with a furnace. Finally, UFG W–Cu composite bulks with a diameter of 20 mm and a height of 10 mm were obtained, which were subsequently used for the following performance tests. For comparison, the commercially available coarse-grained W–Cu composite bulks with a nominal copper content of 30 wt.% were purchased from the market.

### 2.2. Characterizations

The microstructure and morphology were characterized using scanning electron microscopy (SEM, Nova NanoSEM 200, FEI, Hillsboro, OR, USA) equipped with an X-ray energy dispersive spectrometer (EDS) and transmission electron microscopy (TEM, JEOL JEM-2100F, JEOL, Akishima, Japan). The electrical conductivity was measured using the eddy current conductivity meter (Sigma 2008, Xiamen Tianyan Instruments Co., Ltd., Xiamen, China), and all specimens were sanded and mechanically polished prior to testing. The Vickers hardness was measured with a load of 394.2 N and a dwell time of 10 s. At least ten points were measured to obtain reliable data. The room temperature compressive properties were measured using an NKK 4050 universal test machine. The wear resistance was evaluated using the reciprocating sliding tribometer (Lanzhou Zhongke Kaihua Technology Development Co., Ltd., Lanzhou, China). All specimens were sanded and mechanically polished prior to testing. The tests were performed in the air for 30 min with a normal load of 80 N, a rotation speed of 500 r/min, and a travel length of 5 mm. No lubricant was used. A silicon nitride ball with a diameter of 5 mm was used as the counterpart. After the test, the wear scar profile was measured using a profilometer. The high-temperature compression test was performed using a thermal cyclic simulation test machine with a strain rate of 0.02 s^−1^. Thermocouples were soldered to the surface of the specimen for real-time temperature measurements before testing. For the compression process, the specimens were heated to the testing temperature with a rate of 10 °C/s and maintained for 3 min prior to loading. All tests were repeated at least three times to obtain reproducible results.

## 3. Results and Discussion

### 3.1. Microstructures and Properties

**Figure 1:**
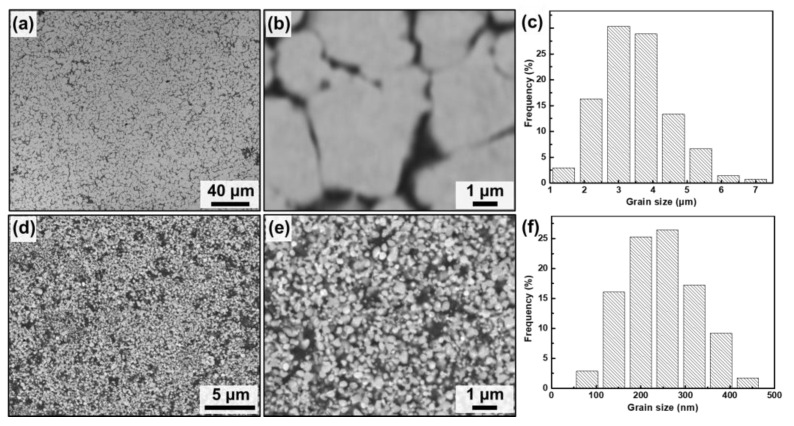
Backscattered SEM images and the corresponding grain size distributions of W in the commercial coarse-grained W–Cu (**a**–**c**) and UFG W–Cu composites (**d**–**f**).

**Figure 2:**
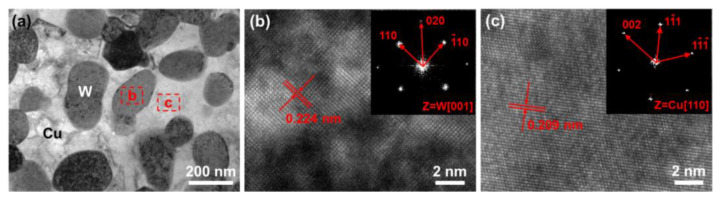
TEM bright field image (**a**) and high-resolution TEM images (**b**,**c**) of the UFG W–Cu composite, inserts in (**b**,**c**) are the corresponding fast Fourier transformation patterns.

The performance of W–Cu composites depends strongly on their microstructures. Figure 1 presents the backscattered SEM morphologies and grain size distribution of the tungsten phase of both the CCG W–Cu composite and the UFG W–Cu composite, where the gray phase is tungsten, and the black phase is copper. As indicated by Figure 1a,b, the CCG W–Cu composite exhibited a uniform microstructure except for a few copper molten pools of several microns. The tungsten phase formed a continuous skeleton structure, meaning a high W–W contiguity. The average grain size of tungsten in the CCG W–Cu composite was ~3.5 μm according to statistics (Figure 1c). This is in accordance with the typical characteristics of W–Cu composites prepared using the conventional infiltrating method, in which a tungsten skeleton was first prepared using sintering, followed by the infiltration of copper at high temperatures, thus leading to the high contiguity and coarse grain size of the tungsten phase [31]. In contrast, as indicated in Figure 1d,f, the UFG W–Cu composite also showed a homogeneous microstructure (the size of copper-rich areas was less than 1 μm), but with a finer grain size (246 nm). This is mainly caused by the short sintering time during SPS. The ultrafine grain size of the tungsten phase was further revealed using TEM (Figure 2). In addition, it can also be found from Figure 2a that the UFG W–Cu composite showed lower tungsten contiguity, which should be attributed to the copper-coated tungsten powders prepared using the electroless plating process [32].

Table 1 shows the hardness, room-temperature compressive yield strength, and electrical conductivity of both the CCG and UFG W–Cu composites. According to the national standard (GB/T8320-2017) for W–30Cu composites, the electrical conductivity and hardness should be no less than 42% IACS and 175 HB, respectively. Thus, both composites meet the criteria for industrial applications. However, as a result of the grain refinement, the UFG W–Cu composite exhibited largely improved Vickers hardness (353 HV) and compressive yield strength (1150 MPa), which were ~78% and ~77% higher than those of the CCG W–Cu composite, respectively. Although, the presence of a large number of interfaces in the UFG W–Cu composite was detrimental to the conductance due to the enhanced interfacial resistance. However, on the other hand, the low tungsten connectivity in the UFG W–Cu composite promoted electron transport [30]. Therefore, the electrical conductivity of the UFG W–Cu composite (49% IACS) was only ~4% lower than that of the CCG W–Cu composites (51% IACS), which was negligible compared to the 77% enhancement in mechanical properties. This is also consistent with the results in our previous study [30], that is, the UFG W–Cu composite prepared in this work exhibits a superior combination of room-temperature mechanical performance and electrical conductivity than the CCG W–Cu composite.

### 3.2. Friction and Wear Resistance

**Figure 3:**
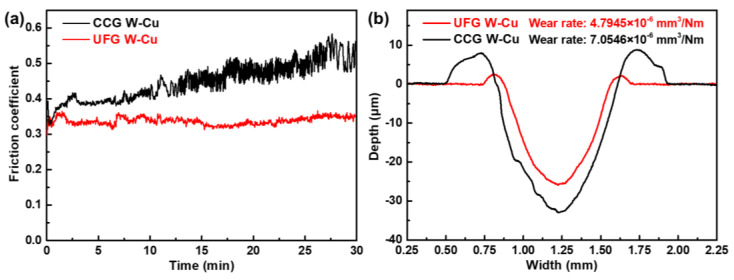
Friction coefficient curves (**a**) and wear scar profiles (**b**) of the CCG W–Cu and UFG W–Cu composites, respectively.

The friction and wear resistance of W–Cu composites greatly affect their service life for use in electrical contact materials or electromagnetic railgun materials. A low friction coefficient and wear rate are always expected. Therefore, the friction and wear behavior of the UFG W–Cu composite and CCG W–Cu composite were investigated and compared. Figure 3a shows the real-time friction coefficient evolution with the friction time of the two composites. It can be observed that the friction coefficient of the UFG W–Cu composite was relatively stable with only a small fluctuation, while that of the CCG W–Cu composite increased gradually with time. The average friction coefficient of the UFG W–Cu composite during the whole wear process was calculated to be 0.34, which has been reduced by 24.4% relative to that of the CCG W–Cu composite (0.45). After the wear test, the profiles of the wear scar were recorded, with the results shown in Figure 3b. For both the UFG and CCG W–Cu composites, significant pile-ups can be found at both ends of the wear scar due to plastic deformation. However, the pile-ups of the CCG W–Cu composite were much larger than that in the UFG W–Cu composite because the UFG W–Cu had higher hardness and greater resistance to deformation [29]. In addition, the width and depth of the wear scar for the UFG W–Cu composite (0.69 mm, 25.65 μm) were also much smaller than that of the CCG W–Cu (0.78 mm, 32.82 μm), indicating a reduced wear rate. The wear rate calculated according to the area of the wear scars for the UFG W–Cu composite was ~4.79 ± 0.16 × 10^−6^ mm^3^/Nm, which has been reduced by 32% relative to that of the CCG W–Cu composite (~7.05 ± 0.35 × 10^−6^ mm^3^/Nm). Evidently, grain refinement contributed significantly to the improved wear resistance of UFG W–Cu composites.

**Figure 4:**
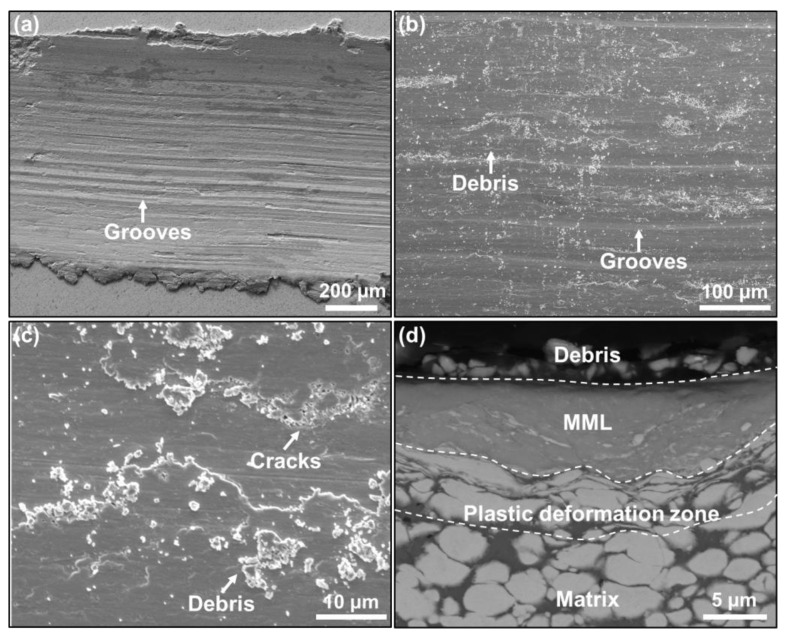
SEM images of worn surfaces (**a**–**c**) and cross-sectional microstructure under the wear scar (**d**) of the CCG W–Cu composite.

**Figure 5:**
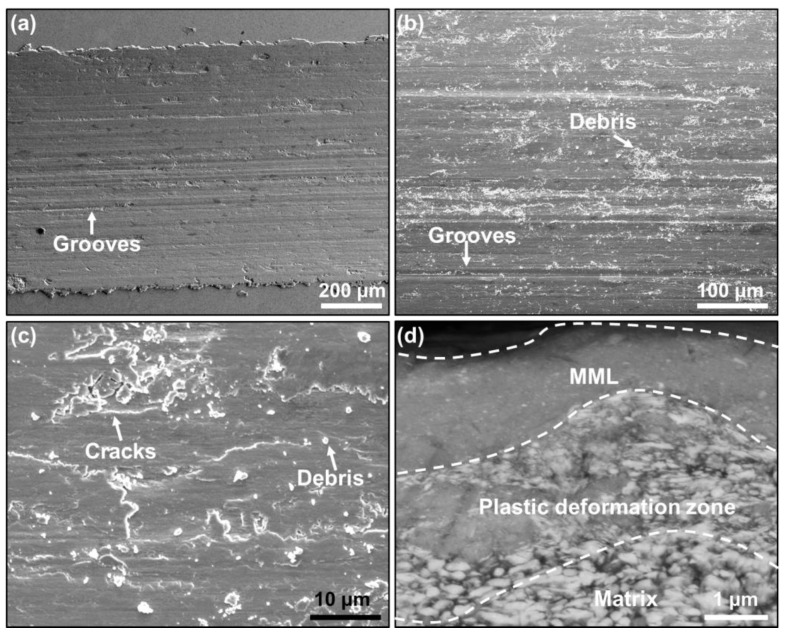
SEM images of worn surfaces (**a**–**c**) and cross-sectional microstructure under the wear scar (**d**) of the UFG W–Cu composite.

In order to shed light on the underlying wear mechanisms and reveal the origin of the low friction coefficient and wear rate in the UFG W–Cu composite, we further investigated the surface morphology and cross-sectional microstructure of the wear scars. As shown in Figure 4a,b, many grooves parallel to the sliding direction and debris were found on the worn surface of the CCG W–Cu composite; that debris can be as large as several microns. In addition, some fatigue cracks were also found on the worn surface (Figure 4c), which were thought to be caused by the continuous reciprocating slide load and would result in the formation of debris [33]. By contrast, as shown in Figure 5a–c, grooves, debris, and fatigue cracks were also found on the worn surface of the UFG W–Cu composite. However, the debris on the UFG W–Cu composite were much smaller than that in the CCG W–Cu composite, which further led to much narrower grooves in the UFG W–Cu composite, since grooves were typically caused by the scratches of the worn debris. It can then be concluded that abrasive wear and fatigue wear are the dominant wear mechanisms for both UFG W–Cu and CCG W–Cu composites, which is also consistent with previously reported results [29].

**Figure 6:**
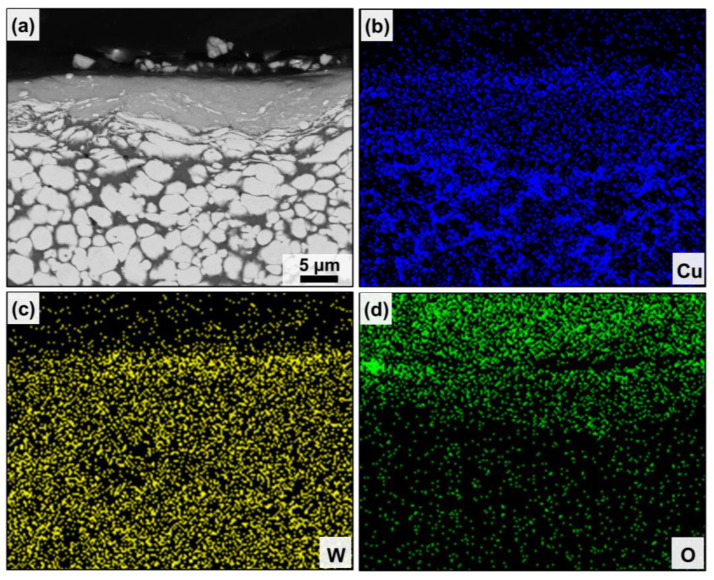
(**a**) Cross-sectional microstructure under the wear scar of the CCG W–Cu composite and (**b**–**d**) the corresponding elemental distributions.

**Figure 7:**
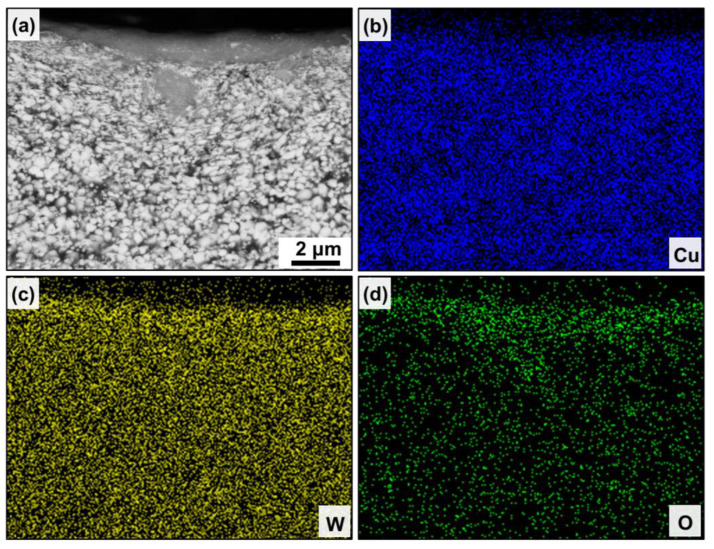
(**a**) Cross-sectional microstructure under the wear scar of the UFG W–Cu composite and (**b**–**d**) the corresponding elemental distributions.

Figure 4d and Figure 5d show the cross-sectional morphology under the wear scar of the CCG W–Cu and UFG W–Cu composites, respectively. As can be observed, three regions can be divided from the interior of the composite to the worn surface: the matrix without deformation, the plastic deformation zone, and the mechanical mixing layer (MML). EDS was used to further confirm the distribution of elements in the three regions, as shown in Figure 6 and Figure 7. It was found that both the matrix and the plastic deformation zone were dominated by tungsten and copper, while a clear enrichment of oxygen was found in the MML. That is, only deformation occurred in the plastic deformation zone, while severe oxidation occurred in the mechanical mixing zone. According to our previous study, the MML was mainly composed of oxides of tungsten and copper [29]. Significantly, the thickness of the MML and plastic deformation zone in the CCG W–Cu composite can be more than 5 μm, which was more than two times that of the UFG W–Cu composite (less than 2 μm).

Hereafter, in order to explain the discrepancy in the wear behavior between the UFG W–Cu and CCG W–Cu composites, an analysis of the wear process was performed on the basis of the observed microstructures. At the beginning of the friction process, the composite was subjected to an additional force from the grinding pair, which was determined using the normal load and the friction coefficient. This created a decreasing stress field in the surrounding region centered around the contact point. Due to the small contact area, the central stress was large enough (exceeding the yield strength) to force the material to deform, and the deformation decreased gradually with the depth. Thus, the plastic deformation region was formed. Thereafter, the surface phase continued to harden under the repeated application of the exerted force, and the break-up finally occurred. Some tungsten particles may also be pulled out. Those fractured and pulled-out metals then spread over the surface of the worn track under the repeated action of friction. Meanwhile, under the action of heat and oxygen, those broken phases oxidized and mixed together to form the MML [34]. In this work, the forces exerted on the surface of CCG W–Cu and UFG W–Cu were initially close since their friction coefficients were close and the normal loads were equal, leading to similar stress fields. However, the UFG W–Cu composite exhibited higher hardness and strength than the CCG W–Cu composite. Therefore, the stress-affected regions, namely the plastic deformation zone and MML, were narrower for the UFG W–Cu composite. For the CCG W–Cu composite, the exerted force was further enhanced with the increase of its friction coefficient, which further increased the thickness of the plastic deformation zone and MML. As the friction continued, under the continuous action of normal and shear stresses, fatigue cracks would occur and propagate in the MML or the interface between the MML and plastic deformation layer, leading to the formation of hard fragments [29], which further resulted in the abrasive wear. Thicker MML in the CCG W–Cu composite tended to produce larger debris, resulting in a high friction coefficient, which in turn led to an increased exerted force and thicker MML. Therefore, it was noticed that the friction coefficient of CCG W–Cu increased with time. Meanwhile, those large fragments exacerbated the abrasive wear, and the thicker MML aggravated the damage caused by fatigue failure, all of which together contributed to the high wear rate of the CCG W–Cu composite. By contrast, the thinner MML in the UFG W–Cu composite enabled smaller debris, which reduced the friction coefficient and weakened the abrasive wear. In addition, the thinner MML also reduced the mass loss rate due to fatigue damage. As a result, the UFG W–Cu composite exhibited a greatly reduced wear rate.

### 3.3. High-Temperature Compressive Properties

**Figure 8:**
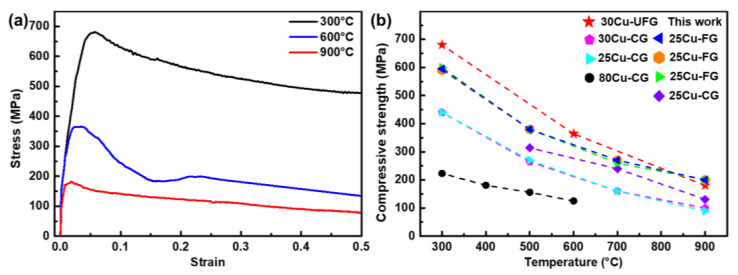
(**a**) Compressive strain–stress curves at different temperatures of the UFG W–Cu composite. (**b**) Comparison of the high-temperature compressive strength between UFG W–Cu and counterparts reported in the literature [12,24,25,27,35,40], where CG and FG denote coarse-grained and fine-grained samples, respectively.

While pure tungsten has excellent high-temperature strength, copper can be extremely soft at high temperatures. It was reported that the strength of copper was less than 30 MPa at 900 °C [35,36]. As a result, the strength of W–Cu composites usually decreases with temperature. However, W–Cu composites have to withstand severe contact-loading at high temperatures in many domains, making high-temperature strength a crucial indicator for applications. Therefore, the compressive behavior of the UFG W–Cu composite was studied. The strain–stress curves of the UFG W–Cu composite at different testing temperatures are shown in Figure 8a. It can be found that the UFG W–Cu composite exhibited a gradually declined compressive strength of ~680 ± 10 MPa, 364 ± 14 MPa, and 180 ± 6 MPa with the temperature raised from 300 °C to 600 °C and 900 °C, which were ~1.55 times, ~1.72 times, and ~1.80 times that of the coarse-grained W–Cu composites. Evidently, grain refinement has greatly enhanced the high-temperature strength of the W–Cu composite. According to the previous study [30], the high strength of UFG W–Cu composites at room temperature was attributed to the load-bearing effect of tungsten and the dislocation strengthening induced by the grain boundaries and phase boundaries. As the temperature was raised, the motion of atoms and dislocations was intensified [37,38], and the critical shear stress required for dislocation slip was reduced [39], so the strength of both copper and tungsten decreased with temperature. Comparatively, however, tungsten remained in a hard phase at high temperatures, making it the dominant load-bearing phase. Moreover, compared with the coarse-grained W–Cu composites, grain refinement enhanced the high-temperature strength of the tungsten phase in the UFG W–Cu composite [37]. Thus, the load-bearing capacity of the tungsten phase was improved in the UFG W–Cu composite. In addition, despite the increased dislocation motion, the largely increased phase interfaces in the UFG W–Cu composite can hinder and accumulate dislocations more efficiently [35]. Therefore, the improved high-temperature strength of the UFG W–Cu composite should be attributed to the enhanced load-bearing effect and dislocation strengthening induced by the grain refinement.

According to previous studies [27,40], the strain–stress curves of both the coarse-grained and fine-grained W–Cu composites at high temperatures could usually be divided into two stages. As the strain increases, their flow stress typically first increases steeply and then rises slowly or remains stable. In addition, they typically exhibit limited plasticity due to the damage of the tungsten skeletons [12]. By contrast, however, our UFG W–Cu composite has shown different high-temperature deformation behavior. The flow stress first increased rapidly to a peak value and then decreased gradually with strain. That is, the strain softening dominated the entire deformation process of the UFG W–Cu composite after yielding. In addition, the UFG W–Cu composite did not exhibit a strain limit during the high-temperature compression test. The above discrepancy is attributed to the finer grain size and the low tungsten contiguity in the UFG W–Cu composite. Dynamic recrystallization is usually considered to be the main reason for strain softening at hot deformation conditions [41]. Furthermore, the determinants of the driving force of dynamic recrystallization are grain boundary energy and dislocation density [42]. For the UFG W–Cu composite, the grain size was much smaller than that of the coarse-grained and fine-grained W–Cu composites, implying a highly improved grain boundary energy. In addition, the increased phase boundaries and grain boundaries in the UFG W–Cu composite can hinder dislocations and improve the dislocation density. As a result, the dynamic recrystallization was accelerated in the UFG W–Cu composite, thus the dynamic softening exceeded the work hardening and dominated. Meanwhile, the low tungsten contiguity in the UFG W–Cu composite enabled a large deformation of the copper phase without causing the fracture of tungsten skeletons, thus contributing to the high strain.

Furthermore, as shown in Figure 8b, we compared the high-temperature compressive strength of our UFG W–Cu composite with that of other binary W–Cu composites [12,24,25,27,35,40]. Typically, both finer grain size and higher tungsten content contribute to a higher strength of W–Cu composites. However, owing to the greatly refined grain size (~240 nm), our UFG W–Cu composite even showed higher compressive strength than the fine-grained W–Cu composites with a copper content of 25 wt.%. In addition, the high-temperature strength of the UFG W–Cu composite was also higher than that of some WC-reinforced W–Cu composites [40]. Although the fine-grained W–25Cu composites with the addition of 3% graphite or carbon nanotubes exhibited higher strength (improved by ~16%) than our UFG W–Cu composite, their electrical conductivity was also severely damaged (decreased by ~46%) due to the formation of a nonconductive WC phase [12,25]. By contrast, the UFG W–Cu composite exhibited a well-balanced high-temperature performance and electrical conductivity, which further highlighted the advantage of grain refinement in improving the comprehensive properties of W–Cu composites.

### 3.4. Evaluation of Overall Performance

**Figure 9:**
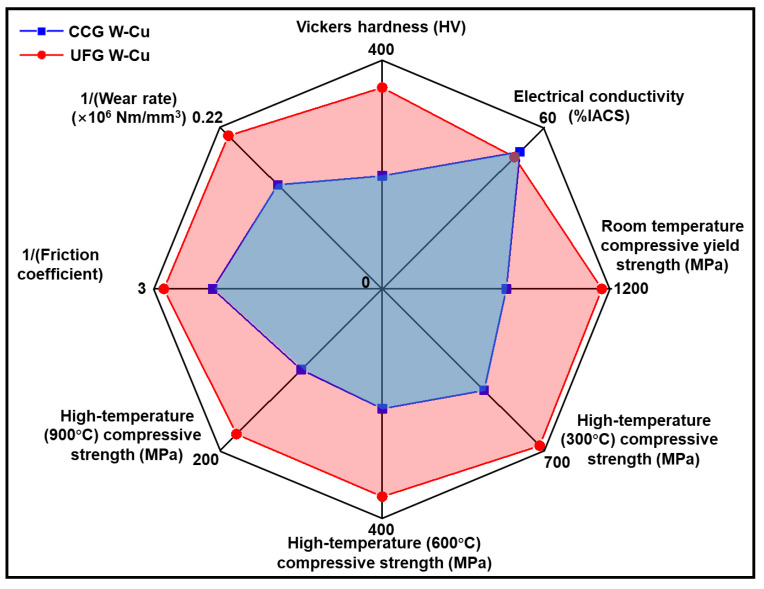
Comparison of the overall properties of the UFG W–Cu and the CCG W–Cu composites, indicating that the UFG W–Cu exhibits superior comprehensive performance.

As we have discussed, the complex service environment of W–Cu composites typically calls for a synergistic improvement of multiple properties rather than the enhancement of a single property. To highlight the performance advantage of the UFG W–Cu composite over the CCG W–Cu composite, a radar map was plotted in Figure 9. The area enclosed by the data points of the UFG W–Cu composite was almost twice that of the CCG W–Cu composite, indicating that our UFG W–Cu composite showed better integration performance than the CCG W–Cu composite. Moreover, unlike those second-phase reinforced W–Cu or nanostructured W–Cu composites, which are strengthened at the expense of conductivity, the UFG W–Cu composite has exhibited substantially enhanced hardness, wear resistance, and room and high-temperature compressive strength compared to the CCG W–Cu composite, with only a negligible decrease in electrical conductivity. It can then be inferred that the UFG W–Cu composite would exhibit better service performance than the CCG W–Cu in some specific domains. For instance, the improved high-temperature strength can effectively increase the maximum welding force and enable the structural stability of the electrodes for resistance welding. This is suitable for the welding of advanced materials with high high-temperature strength, such as superalloys [43]. Additionally, the enhanced wear resistance and high-temperature strength will also mitigate mechanical damage to the surface of guide rails, thus extending the durability of the electromagnetic railgun [44]. Therefore, the UFG W–Cu composite is potentially an alternative to commercial W–Cu composites.

## 4. Conclusions

In this paper, the UFG W–Cu composite with well-balanced room temperature strength and electrical conductivity was prepared using the combination of the electroless plating and SPS techniques. The wear behavior and high-temperature deformation behavior of the UFG W–Cu composite were investigated. It was found that abrasive and fatigue wear were the dominant wear mechanisms for both the UFG W–Cu and CCG W–Cu composites. However, grain refinement enhanced the resistance against plastic deformation, leading to a reduced MML thickness and debris size in the UFG W–Cu composite. This further resulted in the reduced friction coefficient and wear rate of the UFG W–Cu composite. In addition, the high-temperature strength of the UFG W–Cu composite was largely improved due to the grain refinement and enhanced blocking effect of the tungsten phase on dislocations. The ultrafine crystal structure accelerated the dynamic recrystallization, resulting in the strain-softening of the UFG W–Cu composite during high-temperature deformation. Overall, the UFG W–Cu composite exhibits superior comprehensive properties, making it a potential alternative to commercial W–Cu composites.

## Figures and Tables

**Table 1:** Properties of the CCG W–Cu and UFG W–Cu composites.

W–Cu	Hardness/HV_30_	Compressive Yield Strength/MPa	Electrical Conductivity/%IACS
CCG	198 ± 4	650 ± 10	51 ± 0.5
UFG	353 ± 3	1150 ± 8	49 ± 0.5

## Data Availability

The data presented in this study are available on request from the corresponding author.
